# Different patterns of cortical excitability in major depression and vascular depression: a transcranial magnetic stimulation study

**DOI:** 10.1186/1471-244X-13-300

**Published:** 2013-11-09

**Authors:** Carmen Concerto, Giuseppe Lanza, Mariagiovanna Cantone, Manuela Pennisi, Daniela Giordano, Concetto Spampinato, Riccardo Ricceri, Giovanni Pennisi, Eugenio Aguglia, Rita Bella

**Affiliations:** 1 Unit of Psychiatry, Department of Clinical and Molecular Biomedicine, University of Catania, Via Santa Sofia, 78-95123 Catania, Italy; 2 Department “G.F. Ingrassia”, Section of Neurosciences, University of Catania, Via Santa Sofia, 78-95123 Catania, Italy; 3 Department of Chemistry, University of Catania, Viale Andrea Doria 6, 95125 Catania, Italy; 4 Department of Electrical, Electronics and Informatics Engineering, University of Catania, Viale Andrea Doria 6, 95125 Catania, Italy

**Keywords:** Late-onset depression, Cortical excitability, Subcortical vascular disease, Neuroplasticity

## Abstract

**Background:**

Clinical and functional studies consider major depression (MD) and vascular depression (VD) as different neurobiological processes. Hypoexcitability of the left frontal cortex to transcranial magnetic stimulation (TMS) is frequently reported in MD, whereas little is known about the effects of TMS in VD. Thus, we aimed to assess and compare motor cortex excitability in patients with VD and MD.

**Methods:**

Eleven VD patients, 11 recurrent drug-resistant MD patients, and 11 healthy controls underwent clinical, neuropsychological and neuroimaging evaluations in addition to bilateral resting motor threshold, cortical silent period, and paired-pulse TMS curves of intracortical excitability. All patients continued on psychotropic drugs, which were unchanged throughout the study.

**Results:**

Scores on one of the tests evaluating frontal lobe abilities (Stroop Color-Word interference test) were worse in patients compared with controls. The resting motor threshold in patients with MD was significantly higher in the left hemisphere compared with the right (p < 0.05), and compared with the VD patients and controls. The cortical silent period was bilaterally prolonged in MD patients compared with VD patients and controls, with a statistically significant difference in the left hemisphere (p < 0.01). No differences were observed in the paired-pulse curves between patients and controls.

**Conclusions:**

This study showed distinctive patterns of motor cortex excitability between late-onset depression with subcortical vascular disease and early-onset recurrent drug resistant MD. The data provide a TMS model of the different processes underlying VD and MD. Additionally, our results support the “Vascular depression hypothesis” at the neurophysiological level, and confirm the inter-hemispheric asymmetry to TMS in patients with MD. We were unable to support previous findings of impaired intracortical inhibitory mechanisms to TMS in patients with MD, although a drug-induced effect on our results cannot be excluded. This study may aid the understanding of the pathogenetic differences underlying the clinical spectrum of depressive disorders.

## Background

Recently, the finding that patients with late-onset depression had higher rates of brain magnetic resonance imaging (MRI) changes compared with patients with early onset major depression (MD), has led to the hypothesis that mood disorders in the elderly may be related to neurobiological abnormalities, such as cerebrovascular disease [1]. In 1997, Alexopoulos and co-workers [2] introduced the concept of “vascular depression” (VD) as a subtype of geriatric mood disorder characterised by a late age at onset or change in the course of early onset depressive symptoms, persistent symptoms, association with vascular disease or vascular risk factors and diffuse or multifocal cerebrovascular lesions. The “vascular depression hypothesis”, presenting clinically as a depression-executive dysfunction syndrome of late-life, states that disruption of fronto-striatal circuits by vascular lesions predisposes, precipitates, or perpetuates late-life depressive syndromes [3]. Indeed, numerous neuroimaging and neuropathological studies reported increased prevalence and severity of white matter lesions (WMLs) of vascular origin in individuals with elderly depression, especially in those with late-onset illness [4-6].

Nevertheless, depression in the elderly might also result from a recurrent form of MD with an early onset. However, compared with patients with recurrent MD, elderly patients with VD often exhibit a clinical presentation characterized by psychomotor retardation, lack of interest, limited depressive ideation and insight, and prominent disability [7,8]. Moreover, patients with late-onset MD disorder showed specific deficits in attention and executive function [9,10], whereas patients with recurrent MD exhibited deficits in episodic memory [11,12]. These neuropsychological differences are thought to be associated with prominent fronto-striatal dysfunction in late-onset MD, and with a reduction in hippocampal volume in recurrent geriatric MD. The rates of anhedonia and comorbid cardiovascular illness were higher in patients with late-onset MD [11].

Despite a body of literature on clinical, psychopathological, and neuroradiological features of both VD and MD, studies comparing their neurophysiological profiles are lacking. Previous electroencephalography studies in MD patients demonstrated decreased neural activity in the left frontal regions, as shown by an increased alpha power band [13,14]. Recently, changes in transcranial magnetic stimulation (TMS) related-measures of cortical excitability have been shown to be associated with depression. Specifically, most, but not all, TMS studies in patients with MD found reduced activation of both excitatory and inhibitory circuits in the left hemisphere [15-19]. Moreover, some functional neuroimaging studies have shown hypometabolism and hypoperfusion of the left dorsolateral prefrontal cortex (DLPFC) in patients with MD [16,17]. However, the applicability of the findings obtained with TMS on the primary motor cortex (M1) and those obtained with other techniques on the DLPFC requires further investigation.

TMS is a safe and non-invasive neurophysiological investigation technique used to evaluate the cortico-spinal tract, cortical motor areas [20], map motor and cognitive functions, study neural networks, and modulate brain function with a potential therapeutic aim [21-23]. The development of specific stimulation protocols, such as the cortical silent period (CSP) and paired-pulse paradigms, as well as the emerging concept that motor cortical output is influenced by non-primary motor areas, including the ventral and dorsal premotor cortex, supplementary motor area, and cingulate cortex [24], has allowed the use of TMS to explore inhibitory and excitatory interactions within motor cortical regions in several neuropsychiatric disorders [25,26]. A single TMS pulse applied over the M1 through the scalp elicits a motor evoked potential (MEP) in the contralateral target muscles [27,28]. The resting motor threshold (rMT) is believed to reflect membrane excitability of cortico-spinal motor neurons, which is mainly dependent on ion channel conductivity and on excitatory interneurons that project to these neurons [20]. The CSP refers to a suppression of electromyographic activity during a voluntary contraction of the target muscle and depends, at least in part, on inhibitory mechanisms at the level of the motor cortex, probably mediated by gamma-aminobutyric acid (GABA)-b receptors [29]. The paired-pulse TMS couples a suprathreshold magnetic stimulus with a preceding subthreshold stimulus, and the response to the paired stimuli may be increased (facilitation) or decreased (inhibition) depending on the interstimulus interval (ISI): at short ISIs (1–4 ms) the conditioning stimulus determines the intracortical inhibition (ICI) with respect to the test stimulus, whereas at longer ISIs (>5 ms) the effect is intracortical facilitation (ICF). ICI and ICF interactions are likley related to the balance of GABAergic, dopaminergic, and glutamatergic transmissions [29]. In a single study evaluating inhibitory/facilitatory intracortical circuit changes, Bella et al. [30] did not find significant differences between patients with VD and patients with subcortical vascular disease (SVD), suggesting that VD with or without depression might result in a similar neurophysiological profile of cortical excitability, probably as a consequence of cerebral small vessel disease.

In this study, we aimed to assess and compare single and paired-pulse TMS measures of cortical excitability in patients with VD versus MD and between hemispheres. We hypothesised that VD patients would show distinctive neurophysiological changes compared with MD, consistent with the involvement of different neurobiological substrates.

## Methods

### Participants

A sample of 11 patients with VD (6 males and 5 females; mean age, 67.72 ± 3.29 years; mean education, 7.91 ± 5.75 years), 11 drug-resistant recurrent patients with MD without SVD at MRI (5 males and 6 females; mean age, 57.18 ± 7.12; mean education, 12.18 ± 5.98) and 11 age-matched controls (6 males and 5 females; mean age, 67.36 ± 3.75 years; mean education, 9.64 ± 5.08) were consecutively recruited from the Cerebrovascular Disease Center and from the Department of Psychiatry of the University of Catania (Italy).

VD was defined according to the proposed clinical and neuroradiological diagnostic criteria as follows: evidence of vascular risk factors; depression onset after the age of 65 or change in course of depression after vascular disease in people with early-onset depression; presence of some of the following features: cognitive impairment (consisting of, but not limited to, disturbance of executive functions), psychomotor retardation, limited depressive ideation, poor insight, disability, absence of family history of mood disorders, and presence of cerebrovascular disease on neuroimaging [2,31]. VD patients fulfilled the brain MRI criteria for SVD [32], including extending periventricular and deep WMLs or multiple lacunes in deep grey matter, and at least moderate WMLs. VD patients had a primary diagnosis of a current major depressive episode as assessed by the Structured Clinical Interview for DSM-IV-TR Axis I Disorders (SCID-I). They were treated for their vascular risk factors with anti-platelet or anticoagulant medications (aspirin, clopidogrel, warfarin), anti-hypertensive drugs (angiotensin-converting enzyme inhibitors, angiotensin II receptor antagonist, diuretics, dihydropyridine calcium channel blockers), cholesterol lowering medications (statins), and oral antidiabetic drugs or insulin. No patients had focal motor deficits, but a slight reflex asymmetry was present in three patients. Mean age at depression onset in the VD patients was 62.27 ± 5.04 years. No patients were on antidepressant treatment or other psychotropic medicaments.

Drug-resistant MD patients met the DSM-IV-TR clinical diagnostic criteria for recurrent MD as assessed by the SCID-I, and showed a poor response in the course of the current depressive episode (mean duration, 4.33 ± 2.50 months). We defined treatment resistance as drug-resistance to three adequate courses of antidepressants from at least two different classes during the current major depressive episode [33,34].

As shown in Table 1, the pharmacological regimen at the time of the study was: Selective Serotonin Reuptake Inhibitors, Tricyclic Antidepressant, Atypical Antipsychotic Drugs; Serotonin Noradrenaline Reuptake Inhibitors, Tricyclic Antidepressant, Atypical Antipsychotic Drugs. Seven MD patients were on zolpidem. The pharmacotherapy was unchanged throughout the course of the study. Mean age at depression onset in MD patients was 27.82 ± 7.32 years and, including the current episode, 6 patients experienced four major depressive episodes, whereas the remaining 5 patients were diagnosed with five or more episodes.

**Table 1:** Pharmacological treatment of MD patients

**Subject**	**Medication (daily dosage in mg)**
**1**	Paroxetine (20), Nortriptyline (50), Quetiapine (200), Zolpidem (10)
**2**	Paroxetine (20), Nortriptyline (50), Quetiapine (200), Zolpidem (10)
**3**	Sertraline (50), Nortriptyline (50), Quetiapine (200)
**4**	Sertraline (50), Nortriptyline (50), Quetiapine (200), Zolpidem (10)
**5**	Sertraline (50), Nortriptyline (50), Olanzapine (7.5)
**6**	Paroxetine (20), Nortriptyline (50), Olanzapine (7.5), Zolpidem (10)
**7**	Venlafaxine (75), Nortriptyline (50), Quetiapine (200), Zolpidem (10)
**8**	Venlafaxine (75), Nortriptyline (50), Quetiapine (200)
**9**	Venlafaxine (75), Nortriptyline (50), Olanzapine (7.5), Zolpidem (10)
**10**	Venlafaxine (75), Nortriptyline (50), Olanzapine (7.5), Zolpidem (10)
**11**	Venlafaxine (75), Nortriptyline (50), Olanzapine (7.5)

All patients were right-handed, with no history of brain trauma or seizures, and their general and neurological examination was unremarkable. Exclusion criteria were as follows: any non-mood psychotic disorder, chronic medical illness, endocrinopathies other than diabetes associated with depression or affecting cognitive functions (such as thyroid diseases), alcohol or drug abuse, use of drugs causing depressive symptoms (i.e. steroids, beta-blockers, clonidine), Mini Mental State Examination (MMSE) score <24, cases with cortical and/or cortico-subcortical non-lacunar territorial infarcts, borderzone infarcts, haemorrhages, signs of normal pressure hydrocephalus and specific causes of WMLs, and any condition precluding MRI or TMS execution. This study was approved by the local ethics committee based at the “Policlinico-Vittorio Emanuele” University Hospital of Catania (Italy), and all patients provided written informed consent.

### Assessment

All participants underwent a neuropsychological battery including the MMSE as a screening test for overall cognitive impairment, Clinical Dementia Rating Scale for the global cognitive and functional status, Frontal Assessment Battery and the Stroop Color-Word Test interference (normative values were collected from an Italian population sample, Stroop T score, ≤36.92 s; Stroop E errors, ≤4.24) [35] for the evaluation of different frontal lobe abilities, and the 17-item Hamilton Rating Scale for Depression (HRSD-17) for the rating of depressive symptoms. Functional status was evaluated by basic and instrumental activities of daily living (Activity of Daily Living; Instrumental Activity of Daily Living). The physical state of the control subjects was evaluated by general and neurological examinations; their mental state, assessed by means of the SCID-I, was unremarkable. All patients and controls underwent brain MRI scans, acquired using a 1.5 T General Electric system, before inclusion into the TMS study. The protocol included T1-, T2-, proton density-weighted and fluid-attenuated inversion recovery scans; slice thickness was 5 mm with a 0.5 mm slice gap. In the VD group, the severity of deep WMLs was graded according to the visual scale of Fazekas: 0 = absence; 1 = punctuate foci; 2 = beginning confluence of foci; 3 = large confluent areas [36].

### Transcranial magnetic stimulation

MEPs of the right and left first dorsal interosseous (FDI) muscles as well as single-pulse TMS measures of cortical excitability were elicited using a Magstim 200 stimulator (The Magstim Company, Whitland, Dyfed, UK) connected to a 70 mm figure-of-eight coil. The coil was applied with the handle pointing backwards and laterally, at an angle of 45° to the sagittal plane, on the optimum site of stimulation that consistently yielded the largest MEP (“hot spot”). Electromyographic activity was recorded from silver/silverchloride surface active electrodes placed over the motor point of the target muscle, with the reference electrode placed distally at the metacarpophalangeal joint of the index finger. Motor responses were amplified and filtered (bandwidth 3–3000 Hz) with gains of 100 μV and 5 mV/div.

The rMT was defined, according to the IFCN Committee recommendation [37], as the lowest stimulus intensity able to elicit MEPs of an amplitude >50 μV in at least 5 out of 10 trials, with the muscle at rest. The CSP was determined with an approximately 50% of maximum tonic voluntary contraction of the FDI muscles, induced by single TMS pulses delivered at 130% of rMT. The mean CSP duration of five rectified trials was calculated. Central motor conduction time (CMCT) was calculated by subtracting the conduction time in peripheral nerves, estimated by conventional F-wave techniques, from MEP latency obtained during moderate active muscle contraction (10–20% of maximum background force), at a stimulus intensity set at 130% of the rMT [37]. M and F waves were elicited by applying supramaximal electrical stimulation (constant current square-wave pulse of 0.2 ms) to the ulnar nerve at the wrist. The size of MEPs was expressed as a percentage of the supramaximal M wave amplitude (Amplitude ratio). Moreover, to assess spinal motor excitability, the mean amplitude of the F wave was measured in the target muscle. ICI and ICF were studied using the conditioning-test paradigm described by Kujirai *et al. *[38] through a Bistim module (The Magstim Company) connected to a Cambridge Electronic DesignMicro 1401 interface (Cambridge, UK). The procedure consisted of applying two magnetic stimuli in rapid succession through two magnetic stimulators connected to each other. The conditioning stimulus was applied at 80% of the subject’s rMT, and the intensity of the test stimulus was set at 130% of the rMT. The ISIs tested were 1, 3, 5, 7, 10, and 15 ms. Ten trials for each ISI were recorded randomly with an 8-second interval between each trial. The responses were expressed as the ratio of the MEP amplitude produced by paired stimulation to that produced by test stimulation alone. The use of a Bistim module was limited to the paired-pulse TMS measurements only, whereas all other investigations were performed using the single-pulse technique. All measurements were conducted while subjects were seated in a comfortable chair with continuous electromyographic monitoring to ensure either a constant level of electromyographic activity during tonic contraction or complete relaxation at rest. Data were collected on a computer and stored with software ad hoc for off-line analysis [39]. All procedures described above were performed in the same laboratory and situation, by the same operators for each subject at the same time during the day (approximately 3–5 pm).

### Statistical analysis

The non-parametric Kruskal-Wallis ANOVA test was used for comparison of clinical, neuropsychological, and neurophysiological variables obtained from patients and controls (followed by the Mann–Whitney test for post-hoc analysis for the comparison between pairs of groups), and the *χ*2 test was used for categorical variables. The Wilcoxon test for paired data sets was used for the comparison between hemispheres of patients and controls. Nonparametric statistics were used because of the categorical nature of the neuropsychological testing results, and the non-Gaussian distribution of the results of the TMS studies. A *p* value lower than 0.05 was considered statistically significant.

## Results

**Figure 1:**
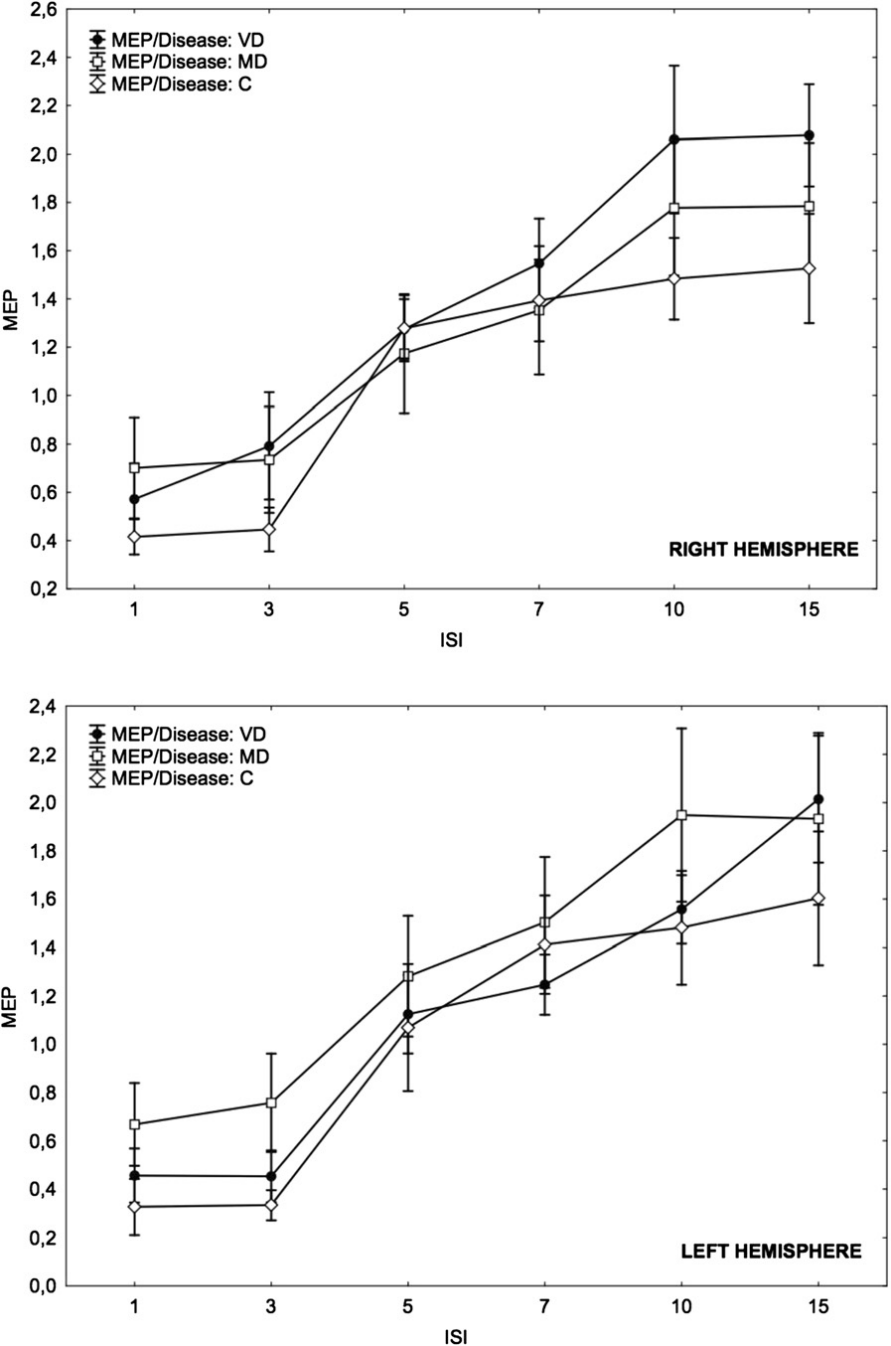
**MEP amplitudes at different ISIs from the 3 groups with respect to the hemisphere.** Data are expressed as mean ± SE. (whiskers). VD = vascular depression; MD = major depression; C = Controls; MEP = Motor Evoked Potential; ISI = Interstimulus Interval.

**Figure 2:**
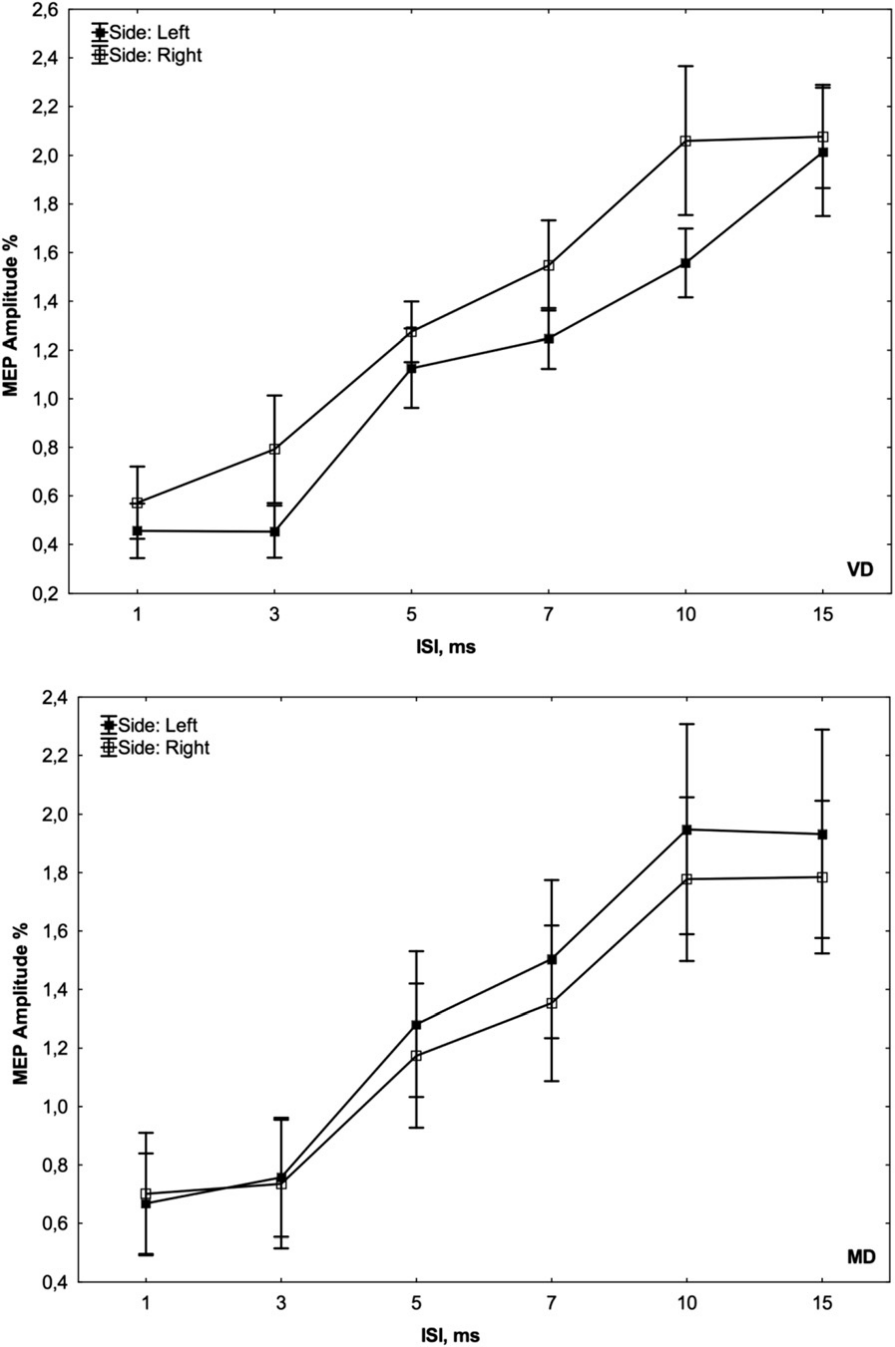
Curves of intracortical excitability obtained from both hemispheres in each group.

The relevant demographic and clinical characteristics of the two patient groups are summarized in Tables 2 and 3. MD were younger than VD patients and controls. The depression rating was less severe in the VD group compared with the MD group. As expected, hypertension was more frequent in VD compared with MD patients, whereas personal history of depressive symptoms was the opposite, and scores at Stroop T were worse in patients compared with controls. WML severity was mild in 4, moderate in 5, and severe in 2 patients. Brain MRI of MD patients and controls was unremarkable (Fazekas 0), and the physical and mental state of controls was also unremarkable. As shown in Table 4, there was a significant increase in rMT in MD patients in the left hemisphere compared with the right (48.36 ± 9.64 vs. 45.00 ± 8.82; p < 0.05). No significant differences were found between the right and left hemispheres for the other measures of cortico-spinal excitability in VD patients, except for a trend toward an increase in ICF at the ISI of 15 ms from the right hemisphere (VD 2.08 ± 0.7 vs. MD 1.78 ± 0.87 vs. Controls 1.52 ± 0.75, p = 0.153) (Figures 1 and 2). No significant differences were found in rMT, central motor conduction time, amplitude ratio, and mean amplitude of the F wave between VD, MD, and controls. In MD patients, the duration of the CSP from both hemispheres was increased, which was significant from the left hemisphere (MD 115.00 ± 32.39 vs. VD 72.18 ± 26.57 vs. Controls 80.09 ± 19.19, p = 0.004), and there was a trend towards significance from the right hemisphere (MD 111.82 ± 42.09 vs. VD 85.45 ± 45.13 vs. Controls 79.18 ± 21.51, p = 0.105) (Table 4).

**Table 2:** Demographic and neuropsychological characteristics of patients and controls

**Variable**	**VD**	**MD**	**Control**	**Kruskal Wallis ANOVA**	
				**H(2.33)**	**p-value**
**Age**	67.72 ± 3.29	57.18 ± 7.12	67.36 ± 3.75	12.20	0.002*
**Age at onset**	62.27 ± 5.04	27.82 ± 7.32	-	-	-
**Education**	7.91 ± 5.75	12.18 ± 5.98	9.64 ± 5.08	3.25	0.197
**MMSE**	26.62 ± 1.58	27.53 ± 2.03	28.36 ± 1.85	5.49	0.064
**ADL**	5.82 ± 0.40	5.81 ± 0.6	6 ± 0	2.00	0.366
**IADL**	7.54 ± 1.21	7.36 ± 1.027	7.82 ± 0.4	1.39	0.498
**HAM-D17**	15.91 ± 7.23	20.27 ± 4.41	4.73 ± 2.41	23.02	0.00001*
**SCID-I**	2.00 ± 0.00	2.00 ± 0.00	-	-	-
**StroopT**	45.70 ± 17.88	34.09 ± 14.37	26.6 ± 11.29	6.82	0.0330*
**StroopE**	2.38 ± 2.71	1.36 ± 1.63	0.94 ± 1.14	1.98	0.370
**FAB**	15.24 ± 2.33	15.12 ± 1.61	16.5 ± 1.78	3.17	0.204

VD = vascular depression; MD = major depression; MMSE = Mini Mental State Examination; ADL = Activity of Daily Living; IADL = Instrumental Activity of Daily Living; HAM-D17 = 17-items Hamilton Depression Rating Scale; SCID-I = Structured Clinical Interview for DSM-IV Axis I Disorders; Stroop T = Stroop Color-Word Test interference score; Stroop E = Stroop Color-Word Test interference number of errors; FAB = Frontal Assessment Battery. Data are expressed as mean ± SD and were analyzed using Kruskal-Wallis ANOVA. * = p < 0.05.

**Table 3:** Clinical characteristics of patients and controls

**Variable**	**VD**	**MD**	**Control**	**Chi-square**	**p-value**
**Gender (male/females)**	6/5	5/6	6/5	0.243	0.886
**Hypertension**	9 (81.8%)	3 (27.3%)	7 (63.6%)	6.947	0.031*
**Atrial fibrillation**	1 (9.1%)	0 (0%)	0 (0%)	2.063	0.357
**Coronaropathy**	1 (9.1%)	2 (18.2%)	0 (0%)	2.200	0.333
**Hypercholesterolemia**	7 (63.6%)	5 (45.5%)	4 (36.4%)	1.699	0.428
**Diabetes**	3 (27.3%)	3 (27.3%)	1 (9.1%)	1.451	0.484
**Familial History**	4 (36.4%)	2 (18.2%)	4 (36.4%)	1.148	0.563
**Personal History**	6 (54.5%)	11 (100%)	2 (18.2%)	15.135	0.001*
**Smoking Habits**	7 (63.6%)	4 (36.4%)	3 (27.3%)	3.226	0.199

VD = vascular depression; MD = major depression. * = p < 0.05.

**Table 4:** TMS parameters of the patient and the control groups obtained from both hemispheres

	**Variable**	**VD**	**MD**	**Controls**	**Kruskal Wallis ANOVA**	
					**H(2.33)**	**p-value**
**Left**	**rMT**	46.00 ± 10.57	48.36 ± 9.64	45.18 ± 6.90	0.451	0.797
	**CSP**	72.18 ± 26.57	115.00 ± 32.39	80.09 ± 19.20	11.182	0.0037*
	**MEP latency**	20.82 ± 2.17	20.28 ± 0.66	20.57 ± 1.73	0.036	0.981
	**CMCT**	6.22 ± 0.97	6.00 ± 1.06	6.19 ± 1.34	0.423	0.809
	**CMCTF**	6.21 ± 0.92	5.33 ± 0.86	6.23 ± 1.28	4.536	0.103
	**A Ratio**	0.40 ± 0.15	0.42 ± 0.13	0.35 ± 0.10	2.786	0.248
	**F Wave A**	0.33 ± 0.50	0.28 ± 0.17	0.13 ± 0.05	3.622	0.163
**Right**	**rMT**	44.00 ± 7.80	45.00 ± 8.82	44.91 ± 5.41	0.035	0.982
	**CSP**	85.45 ± 45.13	111.82 ± 42.09	79.18 ± 21.51	4.503	0.105
	**MEP latency**	20.47 ± 2.06	19.96 ± 1.09	19.71 ± 1.53	0.747	0.688
	**CMCT**	5.82 ± 0.71	5.51 ± 1.05	5.46 ± 0.87	0.872	0.646
	**CMCTF**	5.87 ± 0.76	4.89 ± 0.91	5.45 ± 1.21	5.375	0.068
	**A Ratio**	0.52 ± 0.13	0.35 ± 0.16	0.46 ± 0.12	6.876	0.031*
	**F Wave A**	0.31 ± 0.60	0.10 ± 0.07	0.10 ± 0.04	2.680	0.261
	**Variable**	**Left**	**Right**	**Wilcoxon test**		
				**T**	**p-value**	
**VD**	**rMT**	46.00 ± 10.57	44.00 ± 7.80	11.50	0.192	
	**CSP**	72.19 ± 26.57	85.45 ± 45.13	23.00	0.373	
	**MEP latency**	20.82 ± 2.17	20.47 ± 2.06	19.50	0.230	
	**CMCT**	6.22 ± 0.97	5.82 ± 0.71	16.00	0.130	
	**CMCTF**	6.21 ± 0.92	5.87 ± 0.76	17.00	0.154	
	**A Ratio**	0.40 ± 0.15	0.52 ± 0.13	6.00	0.016*	
	**F Wave A**	0.33 ± 0.50	0.31 ± 0.60	21.00	0.507	
**MD**	**rMT**	48.36 ± 9.64	45.00 ± 8.82	1.00	0.017*	
	**CSP**	115.00 ± 32.39	111.82 ± 42.09	24.00	0.721	
	**MEP latency**	20.28 ± 0.66	19.96 ± 1.09	14.50	0.185	
	**CMCT**	6.00 ± 1.06	5.51 ± 1.05	12.50	0.126	
	**CMCTF**	5.33 ± 0.86	4.89 ± 0.91	13.00	0.139	
	**A Ratio**	0.42 ± 0.13	0.35 ± 0.16	5.00	0.248	
	**F Wave A**	0.28 ± 0.17	0.10 ± 0.07	3.00	0.012*	
**Control**	**rMT**	45.18 ± 6.90	44.91 ± 5.41	19.50	0.722	
	**CSP**	80.09 ± 19.20	79.18 ± 21.51	31.00	0.858	
	**MEP latency**	20.57 ± 1.73	19.71 ± 1.53	3.00	0.007*	
	**CMCT**	6.19 ± 1.34	5.46 ± 0.87	13.00	0.075	
	**CMCTF**	6.23 ± 1.28	5.45 ± 1.21	3.00	0.020*	
	**A Ratio**	0.35 ± 0.10	0.46 ± 0.12	10.00	0.040*	
	**F Wave A**	0.13 ± 0.05	0.10 ± 0.04	10.00	0.138	

VD = vascular depression; MD = major depression; rMT = resting motor threshold (%); CSP = cortical silent period (ms); CMCT = central motor conduction time (ms); CMCTF = central motor conduction time calculated with F-waves (ms); A ratio = amplitude ratio; F wave A = amplitude of F-waves (mV). Data are expressed as mean ± SD. * = p < 0.05.

## Discussion

This is the first report comparing motor cortex excitability between VD and MD patients. The main finding is the observation of potentially distinctive neurophysiological profiles elicited by TMS, adding support to the hypothesis that late onset VD is a different syndrome with respect to early onset recurrent MD. Despite the abundant literature on TMS and depression, only one study has been conducted in VD patients, where VD patients were compared with patients with SVD patients and controls [30]. The results of this study confirm previous findings on VD, and are consistent with most TMS reports investigating MD. An interhemispheric imbalance in frontal cortical activities, as indexed by a higher rMT, was observed in our MD patients and is consistent with an overall hypoexcitability of the left hemisphere [15-17].

In line with our finding of laterality in MD, Soares and Mann [40] described structural asymmetries in cortical and subcortical brain regions crucial in the neuroanatomical model of mood disorders. More recently, it has been reported that decreased relative left-frontal brain electrical activity may be a trait-like marker of depression, suggesting that frontal asymmetry could also be a shared factor predicting first depression onset [41]. However, it remains difficult to infer functional asymmetry between the left and right prefrontal cortices by examining differences in motor responses to TMS only. Findings from previous studies conclude that both glutamatergic and GABAergic pathways may be defective in the left hemisphere of patients with MD. The effect of laterality has also been reported in patients with late-life depression and MRI signal hyperintensities, but it is still controversial as some reports found that left-sided WMLs were significantly associated with older age of depression onset [42], whereas others did not find any laterality effect [43].

Although many, but not all, TMS studies in MD have shown a significant reduction in the amount of both inhibitory (shortened CSP and decreased ICI) and facilitatory (reduced ICF) inputs regarding the left frontal cortex compared with the contralateral hemisphere [17-19], we were not able to find substantial differences. On the contrary, we observed a bilateral increase in CSP duration. The reason for this dissociation remains unclear. Previous studies have obtained conflicting results, probably due to the heterogeneous methods employed, differences in clinical presentation and severity as well as the medication status. For example, Bajbouj *et al*. [17] and Lefaucheur *et al*. [18] found reduced CSP and ICI in medication-free and treated MD patients, respectively. Additionally, Fitzgerald *et al*. [16] did not observe consistent differences in hemispheric activity in drug-resistant MD patients, whereas Levinson *et al*. [19] demonstrated significantly shortened CSP duration in all MD patient groups and decreased ICI in the drug-resistant group only. In contrast, one study observed an increased CSP in MD patients compared with age-matched controls, suggesting increased cortical inhibition [44]. In our study, however, we cannot exclude the possibility of a drug-induced effect on CSP duration, related to zolpidem administration in many MD patients (but not in VD group or controls), highlighting the role of specific subtypes of GABA receptors in the control of inhibitory neuronal loops within the M1 [45]. It is more difficult to explain a drug effect on ICI since it has been shown to be affected by benzodiazepine administration but not zolpidem, and this is consistent with the different segregation of the two inhibitory circuits in the motor cortex at the level of GABA receptor subtypes [46].

Regarding the facilitatory component of intracortical excitability, we observed a trend toward an increase in ICF in VD patients, supporting previous results [30]. A significant hyperfacilitation was found in patients with SVD and clinical features of vascular cognitive impairment-no dementia (VCI-ND) [47], although it was not observed at follow-up [48], supporting the concept that specific TMS measures of cortical excitability can be considered indices of motor cortex plasticity [49]. SVD is indeed a potential aetiology of both VD and VCI and the accumulation of microvascular lesions constitutes a common neuropathological platform for both cognitive decline and depressive episodes in old age [6].

A growing body of evidence indicates that glutamate-mediated compensatory plastic events might also occur in MD [50]. In a recent paper, Spampinato *et al. *[34] demonstrated that repetitive TMS (rTMS) improved performance in a test evaluating frontal lobe abilities and was able to restore inter-hemispheric asymmetry of the ICF and rMT. These results suggested that the TMS changes observed before treatment might be the expression of disruption of glutamatergic receptor plasticity-related processes, and that this neurophysiological behaviour might correlate with executive functions.

Taken together, the data presented here may help to further understanding of the pathogenetic differences underlying the clinical spectrum of depressive disorders. However, relating the distinctive electrophysiological profile between VD and MD patients to what is clinically observed is challenging. Currently, it is difficult to relate the subtle TMS changes with the clinical picture of psychomotor retardation, persistent symptoms, and prominent disability seen in patients with VD. It has been proposed that vascular damage to the reciprocal connections between the prefrontal cortex, basal ganglia, and cerebellum may affect the presentation and the course of late-life VD, although the atrophy of frontal grey matter due to deafferentation could also contribute [10]. Finally, although not statistically significant, we did observe some trends in this study that may be of interest in future studies. For example, the MMSE scores obtained in patients with VD were lower compared with the MD group and controls (p = 0.064), although all values were within normal limits. This could be related to poorer educational level in the VD group, and the clinical presentation and vascular burden. The significance of other trend-level differences, such as for the CMCT from the right hemisphere among the three groups (p = 0.068), and for the CMCT between the left and right side in controls (p = 0.075), is unclear although they may not have a significant clinical relevance, and are likely arising from procedural variability or related to the small samples size in this study.

The findings of this study must be viewed in light of some important limitations. The main limitation is the relatively small number of patients, although they were matched with controls without WMLs that are strikingly prevalent among the elderly. Secondly, given the well-known effect of neuroactive drugs on TMS measures of cortical excitability, we cannot exclude a drug-induced effect on the results. Nevertheless, taking into account these possible interactions, we selected patients assuming psychotropic drugs minimally affected cortico-spinal excitability [51-54], and who were not withdrawn from psychotropic drugs that were unchanged and unmodified in terms of the daily dosage throughout the course of the study. Moreover, medications taken by patients with MD belonged to relatively few classes whose members all share a common mode of action, thus providing more consistent results on the TMS excitability measure [53]. Furthermore, the within- and between-subject variability was minimized, as stated above. The medicaments taken by the VD group for treatment of vascular risk factors (anti-thrombosis agents, anti-hypertensive drugs, statins, oral anti-diabetes therapy) have no supporting data with respect to the possible influence on motor excitability parameters. Motor cortex excitability is unaffected in insulin-dependent diabetic patients when compared with normo- and hyperglycaemic subjects [55], except for a single study reporting a lack of facilitation at an ISI of 30 ms in diabetic patients compared with controls (ISI not explored in the present paper) [56]. Thirdly, transfer TMS findings obtained from stimulation of the M1 to other brain regions, such as those involved in the pathophysiology of depressive disorders, is challenging, and requires further investigation. Finally, although this sample of drug-resistant depressed patients is not likely to be representative of the whole population of patients with recurrent MD, it is homogeneous in the terms of the clinical features and neuropsychological profile.

## Conclusions

The current study showed distinctive patterns of motor cortex excitability between late-onset depression with SVD and early-onset recurrent MD, providing a potential TMS model of the different processes underlying them. These results support the “Vascular depression hypothesis” at the neurophysiological level and confirm inter-hemispheric asymmetry to TMS in MD. Further research and comparison studies with homogeneous groups of patients and other methodologies are needed to confirm the present findings, as well as their modifications over time and clinical correlates.

## Competing interests

The authors declare that they do not have any financial or non-financial competing interests. There was no study sponsor involved in the funding or write-up of this research.

## Authors’ contributions

CC and EA conceived and designed the study and participated in writing the protocol. GL and RR performed clinical and neuropsychological assessments, managed literature searches, and helped to draft the manuscript. MC and MP carried out transcranial magnetic stimulation procedures and analysed the data. DG and CS performed the statistical analysis. GP and RB reviewed the neuroradiological images, drafted the manuscript, and participated in its design and coordination. All authors read and approved the final manuscript.

## Pre-publication history

The pre-publication history for this paper can be accessed here:

http://www.biomedcentral.com/1471-244X/13/300/prepub
